# Quantification of Visual Field Loss in Age-Related Macular Degeneration

**DOI:** 10.1371/journal.pone.0039944

**Published:** 2012-06-29

**Authors:** Jennifer H. Acton, Jonathan M. Gibson, Robert P. Cubbidge

**Affiliations:** 1 Department of Ophthalmology, New York University, New York, New York, United States of America; 2 Ophthalmic Research Group, Optometry & Vision Sciences, School of Life and Health Sciences, Aston University, Birmingham, United Kingdom; 3 Birmingham and Midland Eye Centre, Birmingham, United Kingdom; Massachusetts Eye & Ear Infirmary, Harvard Medical School, United States of America

## Abstract

**Background:**

An evaluation of standard automated perimetry (SAP) and short wavelength automated perimetry (SWAP) for the central 10–2 visual field test procedure in patients with age-related macular degeneration (AMD) is presented in order to determine methods of quantifying the central sensitivity loss in patients at various stages of AMD.

**Methods:**

10–2 SAP and SWAP Humphrey visual fields and stereoscopic fundus photographs were collected in 27 eyes of 27 patients with AMD and 22 eyes of 22 normal subjects.

**Results:**

Mean Deviation and Pattern Standard Deviation (PSD) varied significantly with stage of disease in SAP (both p<0.001) and SWAP (both p<0.001), but post hoc analysis revealed overlap of functional values among stages. In SWAP, indices of focal loss were more sensitive to detecting differences in AMD from normal. SWAP defects were greater in depth and area than those in SAP. Central sensitivity (within 1°) changed by −3.9 and −4.9 dB per stage in SAP and SWAP, respectively. Based on defect maps, an AMD Severity Index was derived.

**Conclusions:**

Global indices of focal loss were more sensitive to detecting early stage AMD from normal. The SWAP sensitivity decline with advancing stage of AMD was greater than in SAP. A new AMD Severity Index quantifies visual field defects on a continuous scale. Although not all patients are suitable for SWAP examinations, it is of value as a tool in research studies of visual loss in AMD.

## Introduction

Age-related macular degeneration (AMD) is the third leading cause of blindness in the world and accounts for blindness in 8.7% of the global population [1]. Due to the increasing elderly population, it is expected that 17.8 million individuals in the US will be affected by 2050 [2]. The functional loss of central vision due to age-related macular degeneration (AMD) is well documented. However of the studies investigating visual field loss in AMD, some reported reduced threshold values [3], [4], [5], [6], [7] whilst others did not [8], [9], [10], [11] and these discrepant findings may reflect differing methods of fundus grading and analyses. In addition no attempt has previously been made to quantify the visual field loss at each stage of severity of AMD. Such knowledge improves our understanding of the natural disease process in terms of visual function and the development of retinal changes. The early identification of patients who would benefit from treatment may help improve visual prognosis.

Past studies evaluating central visual field loss in AMD using non-standard procedures have shown foveal flicker sensitivity to be affected in early AMD [12] and greater flicker perimetry deficits [13]. A number of other techniques have also found functional deficits in AMD, including scotopic sensitivity testing [14], [15], [16], multifocal electroretinogram [17], [18] and preferential hyperacuity perimetry [19]. However these techniques are not readily available for clinical use.

Evidence of short-wavelength sensitivity (SWS) pathway vulnerability in retinal disease suggests the usefulness of short-wavelength automated perimetry (SWAP) in monitoring AMD progression. This is supported by findings of SWS pathway deficits in AMD [20], [21], [22], SWAP sensitivity loss in eyes with soft drusen [6] and in eyes with diabetic retinopathy [23], and the earlier detection of glaucomatous visual field progression by SWAP [24], [25]. Although the use of 30–2 and 24–2 SWAP fields is not clinically widespread due to their greater variability compared to SAP fields [26], the flatter profile of the hill of vision for the 10–2 field allows for more accurate statistical interpretation and greater capability in the detection of focal loss [27].

The aims of the study were to quantify the central visual field loss in a cross-section of AMD patients in standard automated perimetry (SAP) and SWAP. Secondary aims were to evaluate the location of visual field loss in AMD and the appropriate statistical measures that describe visual field loss.

**Figure 1:**
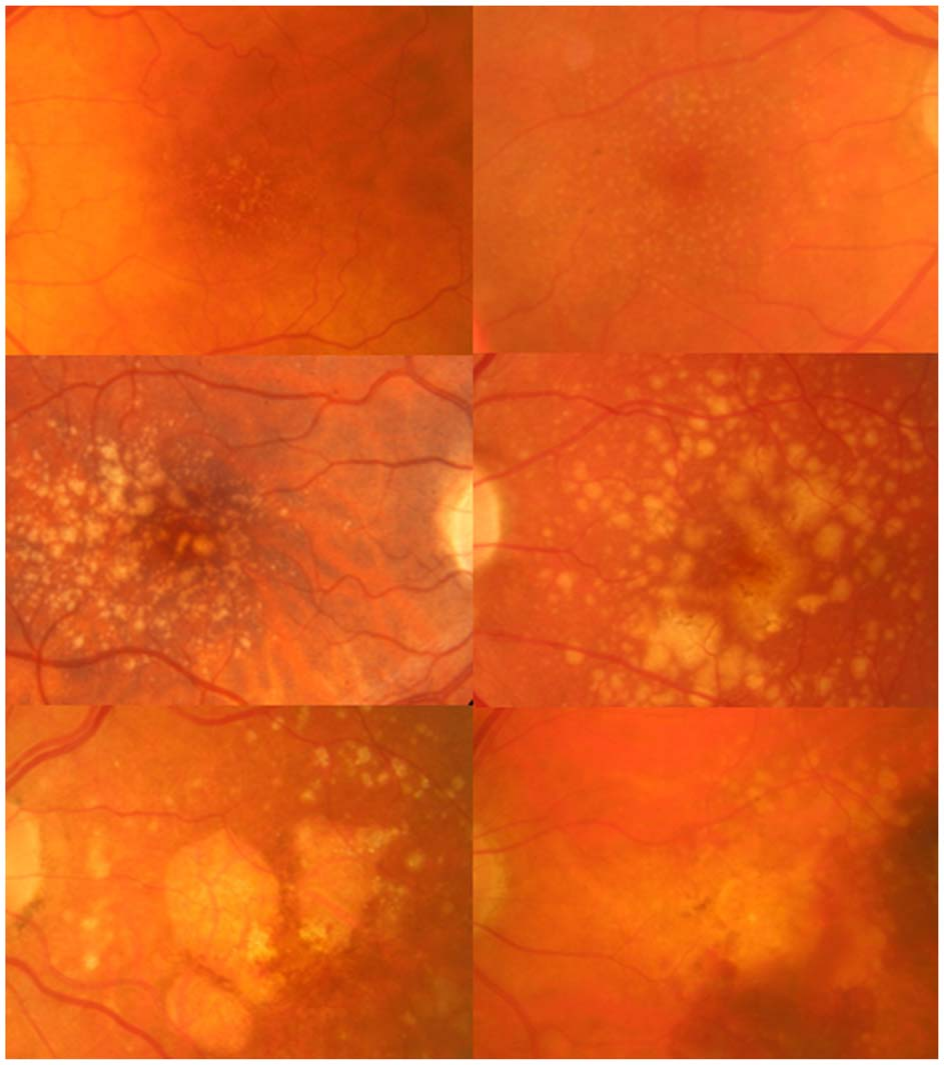
Stages of AMD. Example fundus images of stages of AMD. *Top left*: Stage 0, *top right*: Stage 1, *middle left*: Stage 2, *middle right*: Stage 3, *bottom left*: Stage 4 atrophic AMD, *bottom right*: Stage 4 neovascular AMD.

**Table 1:** Definitions of the Mutually Exclusive Stages of AMD.

Stage	Definition
Stage 0	No signs of AMD, or presence of hard drusen (<63µm) only
Stage 1	Soft distinct drusen (≥63µm) only, or pigmentary abnormalities only
Stage 2	Soft indistinct drusen (≥125µm) only, or soft distinct drusen (≥63µm) with pigmentary abnormalities
Stage 3	Soft indistinct (≥125µm) with pigmentary abnormalities
Stage 4	Atrophic or neovascular AMD

Stages of disease were derived based a longitudinal 6.5 year epidemiologic study of progression rates of AMD [31]. See example images in Figure 1.

## Methods

### Subjects

Subjects were recruited from Birmingham and Midland Eye Centre and the Aston University Eye Clinic. Based on 10° SWAP variability values from the normative database and from a previous study in patients [28], a sample of 7 at each stage would give 80% confidence of detecting a difference of 1.34 dB in mean sensitivity. The sample consisted of 27 patients (mean age 68.8±7.8 years, range 46–84 years, 8 males, 19 females) with a diagnosis of AMD and 22 age-matched healthy controls (mean age 67.2±7.5 years, range 49–78 years, 13 males, 9 females). Subjects had SAP experience but were naïve to SWAP. All study eyes met the inclusion criteria of refraction less than ±5.00 DS and ±2.00 DC, clear ocular media (Lens Opacity Classification System III [29] <NC3, NO3, C1 and P1), no pseudophakia, tonometry <21 mmHg (Pulsair, Keeler, Windsor, UK), normal optic nerve head appearance (assessed by ophthalmologist, JMG), no family history of glaucoma, no history of ocular disease other than untreated AMD, no ocular trauma, no systemic disease, no systemic medication known to influence the visual field, pupil diameters >3 mm and no congenital colour vision defect. Corrected visual acuity was at least 0.1logMAR in each eye, in the normal group and ranged between 0–1logMAR in the patient group. Four patients (of 31 who initially attended) were excluded from the study for non-foveal fixation, as assessed using the ophthalmoscope cross-hair fixation target or due to inability to complete a visual field test.

**Figure 2:**
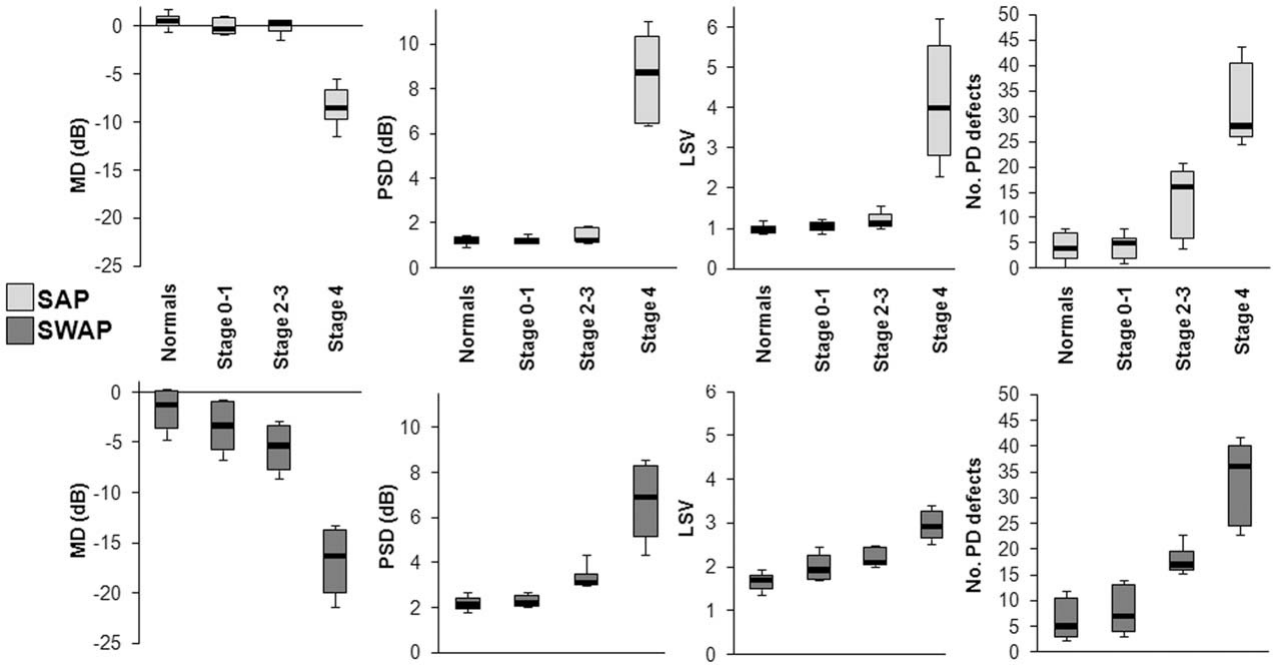
Boxplots representing the change in the visual field with stage of severity of disease. The change in MD (dB), PSD (dB), LSV and number of PD defects as a function of stage of severity of disease, for standard automated perimetry (SAP) and short-wavelength automated perimetry (SWAP) is shown. Boxplot limits represent the 15^th^, 25^th^, 50^th^, 75^th^ and 85^th^ percentiles.

Written informed consent was obtained from each subject and the study had approval from the Aston University Human Sciences Ethical Committee and the NHS West Midlands Research Ethics Committee. The study followed the Tenets of the Declaration of Helsinki.

### AMD Grading

Stereoscopic 30° fundus images were acquired (EOS 10D, 6.3 megapixels, Canon, Tokyo, Japan) and stored as high quality JPEG files (3072×2048) and viewed on a 20.1″ 4is∶3 monitor (1200×1600 pixel resolution), using a prismatic stereoviewer. Image grading [30] and stage of disease [31] (Table 1; Figure 1) was determined in a random order. Subjects who had any gradable features [30] were defined as belonging to the AMD patient group. All grading was determined by independent, masked graders (JMG & JHA), using custom written software (written in Liberty BASIC, Shoptalk Systems, Framingham, MA, USA), which mapped the circular grading grid to the fundus image and incorporated a measurement tool.

### Perimetry

All subjects underwent visual field testing on the dominant eye, as determined by the hole-in-the-card test [32]. Each subject underwent SAP and SWAP 10–2 visual field examinations with the Humphrey Field Analyser 750 (Carl Zeiss Meditec, Dublin, CA, USA) on two occasions. For SAP, the stimulus size was 0.43° (Goldmann III) and the background luminance was 10 cd/m^2^. For SWAP, blue (440 nm) 1.72° (Goldmann V) stimuli were presented on a yellow (>530 nm) 100 cd/m^2^ background. SITA Standard and FASTPAC algorithms were employed for the SAP and SWAP fields, respectively, in order to mimic clinical practice as closely as possible. FASTPAC is the recommended strategy in SWAP [26] and SITA SWAP is not available for the 10–2 field. Visits were separated by 11 days, and the results from the first visit were discarded to account for the learning effect.

**Table 2:** Summary table of global indices as a function of stage of severity of AMD.

SAP							
		MS	MD	PSD	LSV	AMD Severity Index	No. PD defects
Normals	Mean	32.59	0.58	1.21	1.26	0.04	5.09
	SD	1.13	0.98	0.24	0.97	0.04	4.95
Stage 0–1	Mean	32.18	0.05	1.23	1.28	0.04	5.54
	SD	1.25	1.25	0.22	0.91	0.04	5.32
Stage 2–3	Mean	31.41	−0.45	1.39	1.46	0.16	13.86
	SD	1.92	2.10	0.47	0.84	0.15	9.21
Stage 4	Mean	22.47	−9.32	8.72	4.32	0.52	33.14
	SD	4.91	5.18	2.83	1.91	0.14	10.67

SWAP							
		MS	MD	PSD	LSV	AMD Severity Index	No. PD defects
Normals	Mean	24.33	−1.66	2.22	1.71	0.05	6.55
	SD	3.06	2.75	0.40	0.32	0.03	4.42
Stage 0–1	Mean	22.14	−3.72	2.44	2.00	0.08	8.62
	SD	3.77	3.39	0.72	0.37	0.08	6.51
Stage 2–3	Mean	19.71	−5.98	4.10	2.27	0.25	19.29
	SD	4.83	4.67	2.63	0.41	0.16	9.01
Stage 4	Mean	8.64	−16.58	6.69	2.98	0.50	33.43
	SD	4.67	4.32	1.96	0.50	0.17	10.66

Mean sensitivity (MS), mean deviation (MD), pattern standard deviation (PSD), local spatial variability (LSV) and the AMD Severity Index are shown.

**Table 3:** Kruskal-Wallis test of significant variation in visual field measures with advancing stage of AMD.

		MD	PSD	LSV	No. PD Defects	Severity Index
**SAP**	Chi-square	19.630	18.605	17.597	21.032	23.038
	p	<0.001	<0.001	<0.001	<0.001	<0.001
**SWAP**	Chi-square	22.095	26.576	23.878	26.765	28.797
	p	<0.001	<0.001	<0.001	<0.001	<0.001

**Table 4:** Post hoc analyses (Mann-Whitney U test with Bonferroni adjustment) to detect significant differences in visual field measures between AMD stages and normal.

**SAP MD**	Stage 0–1	Stage 2–3	Stage 4		**SWAP MD**	Stage 0–1	Stage 2–3	Stage 4	
	0.129	0.221	**<0.001**	Normal		0.098	0.017	**<0.001**	Normal
		0.905	**<0.001**	Stage 0–1			0.251	**<0.001**	Stage 0–1
			**0.003**	Stage 2–3				**0.006**	Stage 2–3
**SAP PSD**	Stage 0–1	Stage 2–3	Stage 4		**SWAP PSD**	Stage 0–1	Stage 2–3	Stage 4	
	0.785	0.296	**<0.001**	Normal		0.539	**<0.001**	**<0.001**	Normal
		0.321	**<0.001**	Stage 0–1			**0.004**	**0.001**	Stage 0–1
			**0.002**	Stage 2–3				0.025	Stage 2–3
**SAP LSV**	Stage 0–1	Stage 2–3	Stage 4		**SWAP LSV**	Stage 0–1	Stage 2–3	Stage 4	
	0.384	0.039	**<0.001**	Normal		0.042	**0.001**	**<0.001**	Normal
		0.219	**0.001**	Stage 0–1			0.251	**0.001**	Stage 0–1
			**0.004**	Stage 2–3				0.018	Stage 2–3
**SAP No. PD defects**	Stage 0–1	Stage 2–3	Stage 4		**SWAP No. PD defects**	Stage 0–1	Stage 2–3	Stage 4	
	0.744	0.020	**<0.001**	Normal		0.337	**<0.001**	**<0.001**	Normal
		0.067	**<0.001**	Stage 0–1			**0.005**	**0.001**	Stage 0–1
			**0.007**	Stage 2–3				0.013	Stage 2–3
**SAP Severity Index**	Stage 0–1	Stage 2–3	Stage 4		**SWAP Severity Index**	Stage 0–1	Stage 2–3	Stage 4	
	0.313	**0.008**	**<0.001**	Normal		0.183	**<0.001**	**<0.001**	Normal
		0.032	**<0.001**	Stage 0–1			**0.003**	**<0.001**	Stage 0–1
			**0.009**	Stage 2–3				0.013	Stage 2–3

Results show the p-values for the mean deviation (MD), pattern standard deviation (PSD), local spatial variability (LSV), number of pattern deviation (PD) defects and AMD Severity Index. Significant differences are in bold and have a conservative Bonferroni adjustment for multiple comparisons.

**Figure 3:**
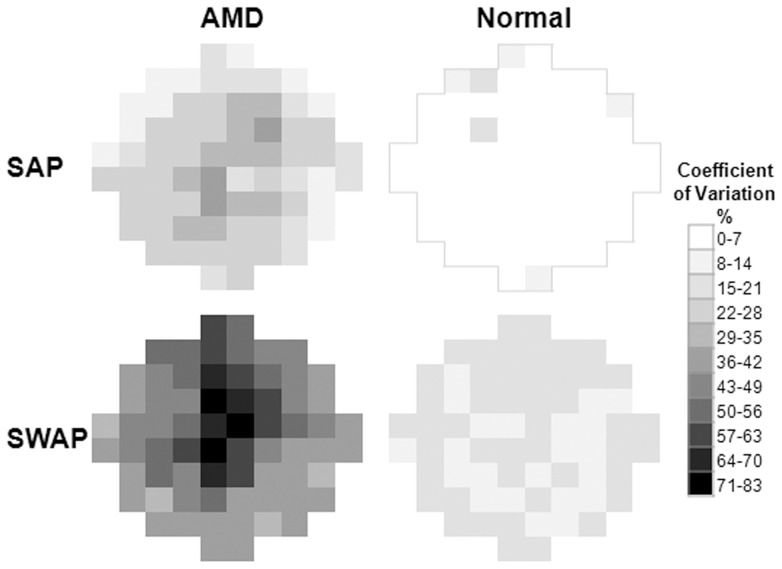
Coefficient of variation (**%**) **map for SAP and SWAP.** Mean coefficients of variation for the AMD patients and normal subjects are shown. Maps are displayed as a right eye.

**Figure 4:**
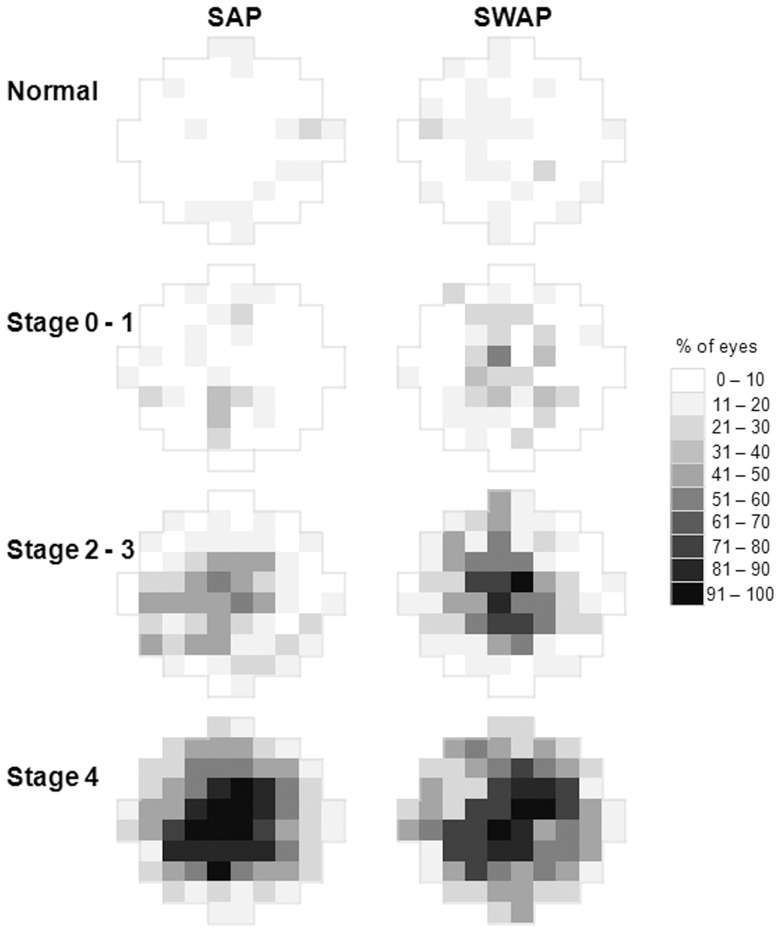
Frequency of defect maps. Frequency of defect maps show the % of eyes at each stimulus location which have significant defects on PD analysis for SAP and SWAP as a function of stage of disease. Maps are displayed as a right eye.

**Figure 5:**
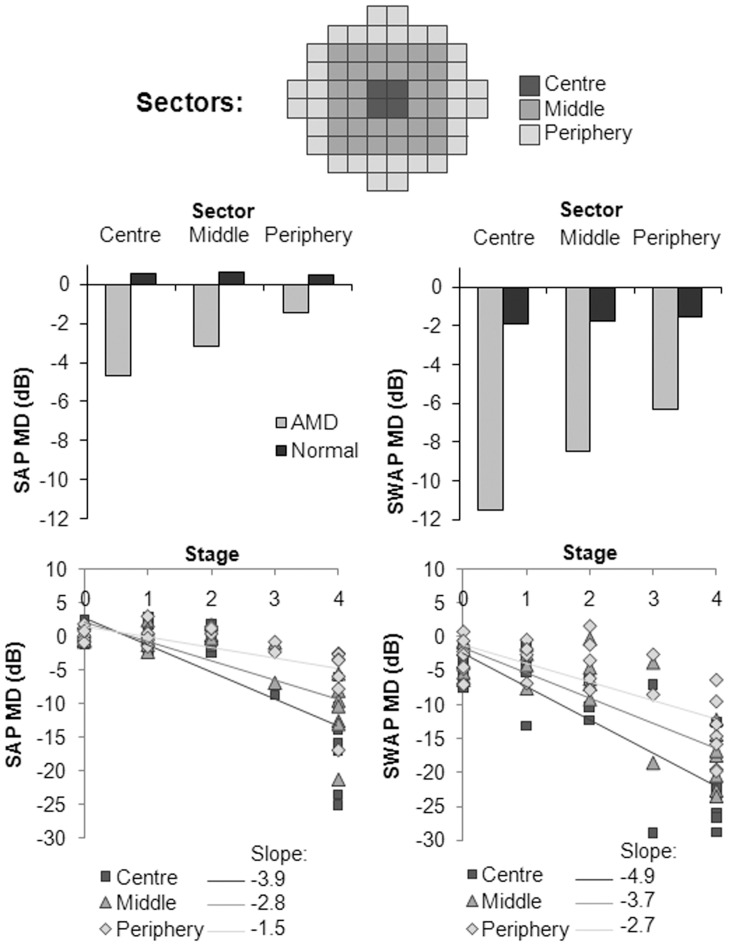
Sector analysis. *Top*: Sector arrangement of the 10–2 stimulus locations. *Middle*: The group mean MD in each sector for SAP and SWAP for normal and AMD subjects is displayed. *Bottom*: The scatterplot of MD as a function of stage of AMD shows the slope of univariate linear regression (dB per stage), in each sector for SAP and SWAP.

**Figure 6:**
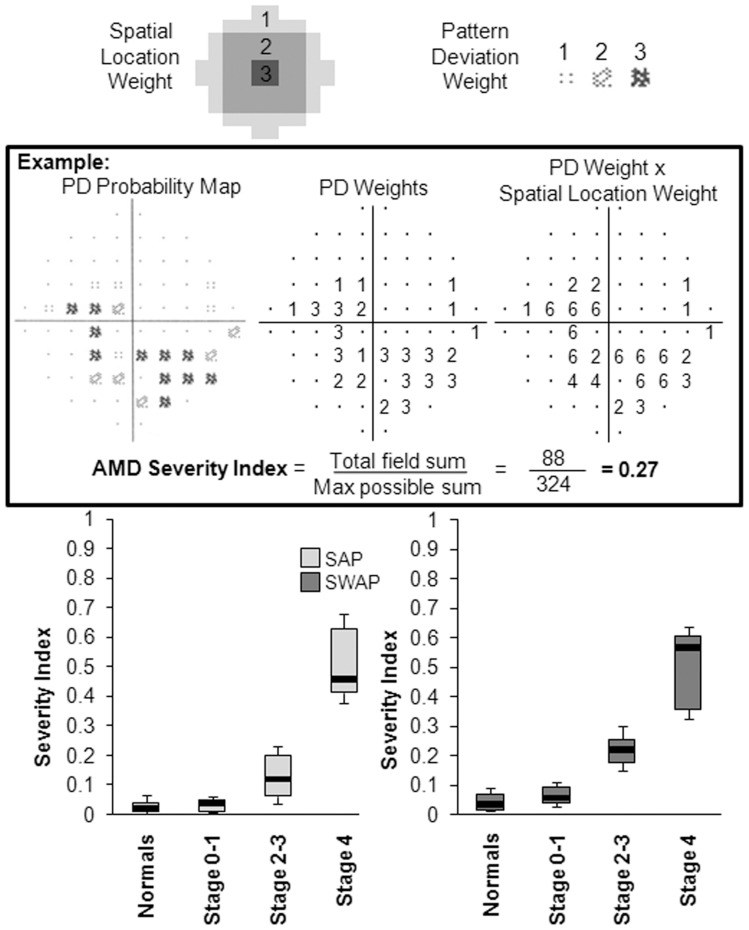
Calculation of Severity Index and change in Severity Index with stage of AMD. Based on Pattern Deviation (PD) maps, visual field sectors were weighted whereby the greatest weight corresponded to the central sector (Spatial Location Weight). This was then multiplied by a depth defect score according to the PD probability value (Pattern Deviation Weight). *Middle, box*: Example calculation: the sum was then divided by the maximum possible score to give the Severity Index ranging between 0 and 1, where 0 indicates no field loss and 1 indicates maximum defects across the entire field. *Bottom*: Boxplots representing the change in Severity Index as a function of stage of severity of disease, for standard perimetry (SAP) and short-wavelength automated perimetry (SWAP). Boxplot limits represent the 15^th^, 25^th^, 50^th^, 75^th^ and 85^th^ percentiles.

**Figure 7:**
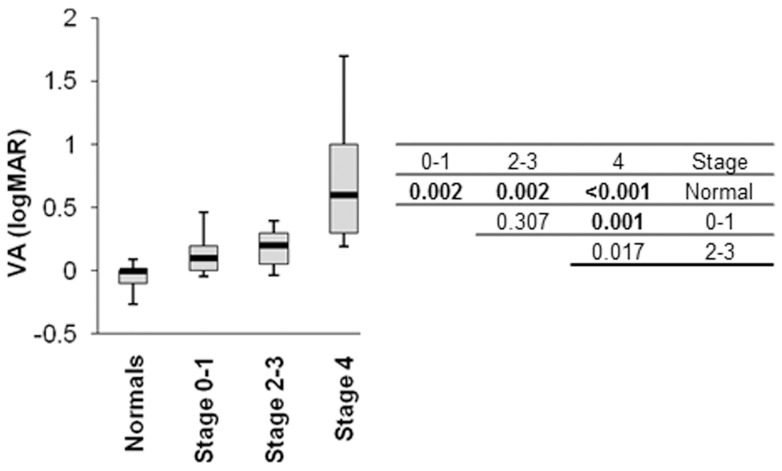
Boxplots representing the change in visual acuity with stage of severity of disease. The change in visual acuity (logMAR) as a function of stage of severity of disease and post hoc analyses (Mann-Whitney U test with Bonferroni adjustment) to detect significant differences in visual acuities between AMD stages and normal are shown. Boxplot limits represent the 15^th^, 25^th^, 50^th^, 75^th^ and 85^th^ percentiles. Significant differences in the posthoc analyses are the p-values in bold and have a conservative Bonferroni adjustment for multiple comparisons.

Before each examination, patients underwent three minutes of background adaptation. Fixation losses, false negative and false positive responses were less than 33%. Regular rest periods were given during and between examinations. The decision to include SAP (SITA standard) fields with false positives >15% and <33% was based on careful assessment of the video monitor gaze tracking function and the other parameters of fixation monitoring.

### Analysis

Visual field change with stage of AMD was determined using the mean deviation (MD), pattern standard deviation (PSD), number of pattern deviation (PD) defects and local spatial variability (LSV). The LSV is a global index calculated as the root mean square of pointwise sensitivity differences between the raw and median filtered visual field [33]. All PD defects <5% were considered significant. The SAP MD and PSD were weighted indices from the HFA printout. Unweighted SWAP indices were calculated from a normal database collected previously. It consisted of 65 normal subjects (age 22–79) who performed 10–2 SWAP visual fields (FASTPAC). The normative data was analysed using univariate linear regression to give the age-corrected normal values at each stimulus location and the confidence intervals were calculated to derive probability defects. The coefficient of variation for each stimulus location was calculated. Frequency of defect maps were constructed from the PD probability maps, which were used in preference to the total deviation analysis as they were less variable and less likely to be affected by the possible presence of diffuse lenticular changes. Concentric sectors of the 10° field, based on the frequency of PD defects, were used to evaluate the unweighted MD and change in MD with stage. We then derived a new index, the AMD Severity Index, using a weighted scoring system based on the PD defects and their locations within the 10° field. The Severity Index was examined for change with stage of AMD.

## Results

Visual field data from 27 eyes of 27 patients and 22 eyes of 22 normal subjects, were included in the analysis. There were 7 eyes graded at stage 0, 6 at stage 1, 5 at stage 2, 2 at stage 3 and 7 at stage 4. Data from stages were combined by the following grouping: stages 0–1, stages 2–3 and stage 4.

### Change in visual field measures with stage

Compared to normal subjects mean sensitivity (MS) values were decreased and SDs were increased in the AMD patients (Table 2). Figure 2 shows the measures MD, PSD, LSV and number of PD defects for normal and AMD subjects. Patients at stages 2–3 and 4 showed a greater variability than those at 0–1 and normal subjects.

All measures varied significantly (Kruskal-Wallis test) with the stage of AMD for both SAP and SWAP (Table 3). A greater number of post hoc differences (Mann-Whitney U test with Bonferroni adjustment; Table 4) were noted between subject groups for SWAP than for SAP, for the PSD and the LSV. Overall, SWAP detected more differences from normal than SAP. In SWAP, the MD had the fewest significant differences from normal. In SAP more significant differences from normal were evident for the Severity Index.

There were significant correlations between LSV and PSD for SAP (Spearman's rho = 0.455, p = 0.001) and SWAP (rho = 0.768, p<0.001).

### Coefficient of Variation

As expected, the AMD group yielded lower MS and greater SDs than normal group for SAP and SWAP (Table 2). Variability was compared using the coefficient of variation statistic [26], [27] (SD/mean, expressed as %), which represents a normalised measure of dispersion such that distributions which differ in the magnitude of their measurement scales may be compared.

SWAP consistently revealed greater coefficients of variation than SAP (Figure 3). The AMD patients had higher values than normal. At AMD stages 0–1, mean coefficients of variation were only slightly greater than normal (normals: SAP 5±3% SWAP 16±2%; Stage 0–1: SAP 6±3%, SWAP 21±5%) but at stage 4 large values were exhibited (SAP 44±20%; SWAP 105±89%).

### Frequency of Defect

The frequency of defect maps (Figure 4) represent the percentage of eyes at each stimulus location with PD defects. SWAP defects occurred more frequently than SAP defects in early AMD. For late stage AMD (stage 4), for both perimetry types, 70–80% of eyes had defects within the central 5°. Enlargement of a central scotoma was noted with progressing stage of disease. The increase in frequency of defects with worsening disease stage occurred at an earlier stage in SWAP than in SAP.

### Sector Analysis

Based on the frequency of defect maps, the field was divided into the following sectors: centre, middle and periphery (eccentricities: 1.4°, 3.2–7.1° and 7.1–9.1° respectively; Figure 5 *Top*). For AMD patients, central sector MD values were worse than the peripheral values for both SAP and SWAP (Figure 5 *Middle*). For the normal group, MD values remained uniform across all sectors. Figure 5 (*Bottom*) shows the regression slope of MD as a function of stage for each sector for SAP and SWAP. The most rapid sensitivity loss occurred in the centre and the slowest in the periphery.

### AMD Severity Index

Based on our sector analysis and frequency of defect maps, a Severity Index of AMD visual field loss was derived (Equation 1) [34]. Visual field sectors were weighted by their location within the 10° field and multiplied by a depth defect score according to the PD probability value (Figure 6). Centrally located defects and more severe probability values carried a greater weight. The sum was then divided by the maximum possible score to give the Severity Index ranging between 0 (no field loss) and 1 (maximum defects across the entire field).
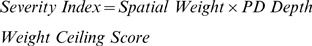
(1)


A larger Severity Index (Figure 6 *Bottom*) in SWAP than in SAP for each AMD group indicated deeper and more extensive sensitivity loss in SWAP. Severity scores varied significantly with stage of disease for SAP and SWAP (Table 3). Post hoc analysis (Table 4) revealed significant differences between normal subjects and AMD patients at stages 2–3 and 4, in SAP and in SWAP.

### Visual Acuity

Figure 7 shows the visual acuities in the normal subjects and AMD patients. Visual acuities varied significantly with stage of AMD (Kruskall-Wallis test: Chi-square = 28.09, p<0.001). A greater number of post hoc differences from normal (Mann-Whitney U test with Bonferroni adjustment; Figure 7) were found than for perimetry.

## Discussion

In this cross-sectional study of patients with AMD, sensitivity loss in 10–2 SAP and SWAP visual fields increased with increased severity of disease. The finding of reduced MS and greater variability in sensitivity in the AMD patient group compared to the normal group is consistent with previous studies [3], [4], [5], [6], [9].

We quantified the visual field loss as a function of stage of AMD and found that the changes in SWAP were more significant than those in SAP. SWAP had a greater capability in detecting differences between the AMD groups and differences from normal, than SAP. Previous studies of blue-on-yellow perimetry in AMD [5], [6] did not compare results to SAP, however in patients with diabetes, sensitivity loss in the 10–2 visual field, due to structural change, was better detected by SWAP than SAP [23], [35], [36], [37]. The SWS pathway is vulnerable to a variety of retinal disease implying that SWS pathway sensitivity loss occurs at multiple sites of damage [38]. Possible explanations include reduced redundancy due to the sparse SWS system [39] and/or the smaller response range of the SWS cone system [40].

A secondary aim was to examine the location of visual field loss within the 10° field. A central scotoma within 5° was demonstrated in late stage AMD, supportive of previous findings of reduced SWS in the central compared to the peripheral 10° field in AMD [5]. Others have indicated paracentral scotoma and preservation of central vision in AMD [41], [42], or no difference between central and peripheral sensitivity in the 10° SWAP field [6]. Possible explanations for these varied results include differences in instrumentation, sample sizes, classification of AMD and analyses.

In fact, we found that the most vulnerable region to AMD related sensitivity loss was the central 1°, in which the change was −3.9 dB per stage in SAP and −4.9 dB in SWAP. Our results suggested a symmetrical defect, from which the Severity Index was derived, to classify visual field defects on a continuous scale. In SAP, the Severity Index detected the most differences between groups. Thus, it may be a useful method for monitoring longitudinal progression of the visual field in AMD. From our findings, a Severity Index >0.1 in SAP and >0.2 in SWAP appeared abnormal, however a larger study is required to investigate this.

All visual field measures varied significantly with increasing stage of disease, however there was some functional overlap between stages. We found that focal measures of SWAP visual field loss, such as the PSD and number of PD defects, demonstrated greater capability in detecting AMD subjects from normal. In a previous study [23], detection of focal loss by the number of PD defects in SWAP, had a stronger relationship with diabetic retinopathy than SAP.

Of the focal indices, LSV is less common but statistically less manipulated than PSD, as well as significantly correlated with PSD. LSV is less influenced by pre-receptoral absorption, i.e. macular pigment and lenticular changes, which limit the interpretation of SWAP. Although the effect of lenticular absorption was minimised by our exclusion criteria, mild absorption and macular pigment effects may have influenced the SWAP data. However, significant attenuation of SWAP thresholds by macular pigment was not evident from normal defect maps (Figure 4), nor in our normal database. Correcting for macular pigment, which has a high within-subject variability [43], would artificially increase perimetric sensitivities and alter normal prediction limits. Since the effects of pre-receptoral absorption on SWAP are symmetrical and diffuse, a statistical approach has been recommended to separate focal loss [43], [44] and our results support the conclusion that focal loss is of greater importance in AMD.

A limitation of SWAP was that overall larger coefficients of variation were found than in SAP (Figure 3), similar to previous findings [26]. However, this finding was skewed by large values at stages 3 and 4, where greater variability would be expected in scotomatous locations. In fact, coefficients of variation at earlier stages were near normal for both perimetry types, indicating SWAP as a reliable method to examine early stage AMD patients with minimal lens opacities.

In our study SWAP analysis had a high correlation with visual acuity as a means of detecting AMD and this raises the question as to what extra value SWAP measurement adds in assessing patients with AMD. It is known that central perimetry in AMD gives information about the spatial extent and depth of central visual loss that visual acuity cannot measure [45], and with increasing possibilities of medical intervention available, this aspect of visual function measurement is likely to become important. In fact, visual acuity appeared to better differentiate between early stage AMD and normal, than perimetry. Therefore the value of SAP and SWAP analysis in AMD is in research, rather than as a diagnostic tool.

Limitations of the study were the small number of eyes at individual stages, which led to the grouping of stages and the cross-sectional design of the study. The difference in SAP and SWAP normative databases limits the analyses, however use of the HFA Statpac analysis is standard in clinical practice and provides greater accuracy than collection of new SAP normative data. A longitudinal study in a larger sample, following visual field progression over several years is warranted, in which the Severity Index could be used to measure field defects on a continuous scale. In a larger sample it would be possible to differentiate results between late stage patients with geographic atrophy and choroidal neovascularisation. Specific consideration in a progression study, would be necessary to account for worsening cataracts and potential involvement of fixation later in the disease, which may affect the interpretation of visual field results. Furthermore, visual field testing may be useful in assessing the effectiveness of novel treatments in either preserving or improving visual field loss in patients with AMD, enrolled in a clinical study under appropriate exclusion criteria. Due to the strict exclusion criteria of our study, our findings are limited to AMD subjects suitable to visual field testing, i.e. those who have foveal fixation and clear ocular media.

The results present evidence of a relationship between the SWAP visual field and severity of AMD, whereby sensitivity declined with advancing stage of AMD. SWAP had greater capability in detecting early AMD than SAP. The importance of early detection of functional change in AMD is clinically relevant to possible earlier intervention or lifestyle changes. Sensitivity loss in AMD was focal in nature and the central field became less uniform as stage increased. SWAP defects occurred at similar locations but were deeper and wider than corresponding SAP defects. Although not all patients are suitable for SWAP examinations, our findings support it as a useful tool in research studies of visual loss in AMD.
